# Microbial Aerosols Generated from Standard Microbiological Laboratory Procedures

**DOI:** 10.1089/apb.2021.0038

**Published:** 2022-05-27

**Authors:** Thomas Pottage, Didier Ngabo, Simon Parks, Helen Hookway, Neville Q. Verlander, Kazunobu Kojima, Allan M. Bennett

**Affiliations:** ^1^ Research and Evaluation, UK Health Security Agency, Salisbury, United Kingdom.; ^2^ Statistics, Modelling and Economics Department, UK Health Security Agency, London, United Kingdom.; ^3^ World Health Organization, Geneva, Switzerland.

**Keywords:** laboratory procedures, aerosol generation, pipetting, spillage, bacterial spore

## Abstract

**Background:**

Modern microbiology laboratories are designed to protect workers and the environment from microbial aerosols produced during microbiological procedures and accidents. However, there is only limited data available on the aerosols generated from common microbiology procedures.

**Methods:**

A series of common microbiological procedures were undertaken with high concentration spore suspensions while air samplers were operated to sample the aerosols generated. Surface contamination from droplets was visualized using sodium fluorescein within the suspension. A total of 36 procedures were studied using different sample volumes (0.1–10 mL) and two spore suspension titers (10^7^ and 10^9^ colony forming units [cfu]/mL).

**Results:**

The aerosol concentrations generated varied from 0 to 13,000 cfu/m^3^. There was evidence to suggest that titer, volume, and poor use of equipment were significant factors in increased aerosol generation from some of the procedures. A risk assessment undertaken using the data showed that any aerosol generated from these processes would be contained within a correctly operating biological safety cabinet. Therefore, with these procedures, the operator and the environment would not require any additional protective measures such as respiratory protective equipment or a negative pressure laboratory to prevent aerosol exposure or release.

**Conclusions:**

Aerosol generation from common laboratory processes can be minimized by reducing sample volumes and concentrations if possible. Training laboratory staff in good microbiological techniques would further mitigate aerosols generated from common laboratory processes.

## Introduction

The modern microbiology laboratory is designed to prevent exposure of staff to microbial aerosols by containing potential aerosol generating processes within biological safety cabinets (BSCs) utilizing directional air flow and high efficiency particulate air (HEPA) filtration.^1,2^ The laboratory itself is often operated at a negative pressure to prevent release of aerosols into the outside environment. In higher containment laboratories, extract HEPA filters are used, and in the highest level of containment, laboratories may be sealed to prevent release of agent in case the extract filter system malfunctions.

In other situations, laboratory staff may wear respiratory protective equipment (RPE) to protect themselves against aerosols as well as using safety cabinets. All these systems can be validated, tested, certified, and protection values and efficiencies assigned.^3,4^ However, the potential exposure of workers to microbial aerosols in modern microbiological facilities for a range of tasks is generally unknown, therefore, it is difficult to complete a quantitative risk assessment to ensure the containment measures used are appropriate for the aerosol infection risk.^5^

The concentration of microbial aerosols in microbiology laboratories has been measured before. In the 1960s and 70s, Kenny and Sabel,^6^ and Dimmick et al.^7^ quantified the microbial aerosol generated during common microbiological procedures of their time, which included the use of platinum loops and other practices no longer used. Ashcroft and Pomeroy measured aerosol concentrations generated by catastrophic fermenter failures.^8^ Bennett and Parks measured aerosols generated from accidents in the microbiology laboratory,^9^ and in a more recent article, Pottage et al. studied pipetting and plating out and the effect of training on reducing aerosol generation.^10^

In the years since the Kenny and Sabel, and Dimmick studies, the microbiology laboratory has changed markedly in two main ways. First, there has been a major change in the methods and technologies used in the microbiology laboratory. Rapid diagnostic methods, such as polymerase chain reaction and sequencing technologies, have replaced traditional culture assays as first line diagnostics tools. Some of the biochemical assay tests used in the past with 10 mL test tubes have mostly been replaced by API strips using lower volumes, and then subsequently been replaced by simple methods utilizing colony picks such as matrix-assisted laser desorption/ionization-time of flight.^11^

As a result, the amount of work undertaken with pathogenic micro-organisms has reduced, and lower volumes and titers are used in combination with intrinsically safer processes. Second, the equipment used in microbiology laboratories is designed to be safer and more contained. Automatic pipettes are now widely used with much smaller volumes of infectious materials, and potentially aerosol-generating procedures such as centrifugation and homogenization occur in contained devices.^12^ HEPA filters are more reliable and BSCs are better understood and regularly checked and serviced.^13^ The high percentages of laboratory infections caused by aerosolization reported by Pike and Collins have not been reported by recent reviews of laboratory infection.^14,15^

The objective of this study is to quantify the aerosol generation potential of a range of techniques currently used in the microbiology laboratory using different volumes and concentrations of a bacterial spore tracer while the air is sampled close to the procedure. The data obtained are then used to carry out risk assessments of the potential microbial aerosol exposure of those working in the laboratory and outside. Providing an evidence base for biosafety practices is a way of ensuring that any exposure is prevented with appropriate control measures without entailing any unnecessary expenditure.^5^

## Materials and Methods

### Biological Tracer/Fluorescein Solution

*Bacillus atrophaeus* spores (NCTC 10073) were used as biological tracer. A *B. atrophaeus* spore suspension was diluted in sterile distilled water (SDW) to produce a working stock suspension of 1 × 10^9^ colony forming units per milliliter (cfu/mL). The stock suspension was heat shocked at 70°C for 20 min in a shaking water bath, cooled to room temperature, and the concentration confirmed by serial dilution then enumerated on Trypticase soy agar (TSA; Oxoid, United Kingdom) postovernight incubation at 37°C.

A separate 10^7^ cfu/mL suspension of *B. atrophaeus* was produced from the 10^9^ cfu/mL suspension by dilution in SDW. Both spore suspensions were stored at 4°C. Sodium fluorescein salt powder (Sigma, United Kingdom) was added to both 10^7^ and 10^9^ cfu/mL *B. atrophaeus* spore suspensions at 0.01% concentration to allow quantification of suspension splashes on surfaces after each test was completed through visualization of droplets (yellow under UV light; 395–400 nm wavelength, UV LED flashlight, LemonBest Bright, China).

### Air Sampling

Two Sartorius MD8 sampler heads (Sartorius, Germany) connected to separate vacuum pumps (diaphragm pump; KNF Neuberger, Germany) were set up in a Class II cabinet at 12 cm above the work surface and ∼30 cm apart on clamp stands. Both sampler heads were fitted with gelatine membrane filters (Sartorius, Germany) and the air flow rate was measured with a calibrated Thermal Mass Flowmeter (TSI 4000 series). The rear MD8 ran at 31 L/min and the front MD8 at 48 L/min, sampling a total volume of 155 and 240 L, respectively, in a 5 min period.

The cabinet was vented for a minimum of 5 min before each test to eliminate residual airborne contamination. Cabinet ventilation was then turned off for the duration of each test run to allow capture of aerosol by the air sampler (5 min). Background samples (5 min) were taken before and after each set of test procedures to determine the baseline aerosol level. After each test run, gelatine membrane filters were recovered from Sartorius MD8 sampler heads with sterile disposable tweezers (SLS, United Kingdom), placed directly on TSA and colony counts enumerated after incubation for 24 h at 37°C.

### Experimental Procedures

The detailed experimental procedures are described in the Supplementary Data, an overview of the parameters for each procedure are given in Table 1.

**Table 1.** Overview of the parameters that were used for each test during (full procedural details in Supplementary Data)

Procedure	Container/technique	Volume (mL)	Suspension concentration (cfu/mL)
Pipette mixing	96-Well plate	0.1	10^7^ and 10^9^
	Cryotube	0.1	10^7^ and 10^9^
	Universal	1, 10	10^7^ and 10^9^
Serial dilution	96-Well plate	0.1	10^7^ and 10^9^
	Cryotube	0.1	10^7^
	Eppendorf	0.1	10^7^ and 10^9^
	Universal	1, 10	10^7^ and 10^9^
Vortex mixing	Eppendorf	1	10^7^ and 10^9^
	Universal	1, 10	10^7^ and 10^9^
Handshake	Eppendorf	1	10^7^ and 10^9^
	Universal	1, 10	10^7^ and 10^9^
Plating	Blue loop	0.1	10^7^ and 10^9^
	Spreader	0.1	10^7^ and 10^9^
Pi pump	Normal operation	5, 10	10^9^
	Valve release	10	10^9^
Pipette boy	—	5, 10	10^9^
Colony pick and emulsify	—	—	10^9^
Eppendorf flick open	—	1	10^7^ and 10^9^
Bead blaster and open	5000 rpm	1.4	10^9^
Accident	Knock over	5	10^9^
	Tube drop	5	10^9^
Tissue grinder	With plug	5, 10	10^9^
	Without plug	5, 10	10^9^
Plate sniff	—	—	10^9^

### Statistical Interpretation

The measured airborne concentration in cfu per cubic meter (cfu/m^3^) of each MD8 sampler head in each test procedure was calculated using the following formula: cfu/m^3^ = cfu count ÷ (flow rate [m^3^] × air sample time [min]). The cfu/m^3^ from each test replicate run was averaged from the front and back sampler results, after which the study data were analyzed. The average count from surface contamination data represented by fluorescein splash on hands and Benchkote was calculated from triplicate runs in each test procedure.

A multilevel ordinal logistic regression model with test as the random effect or a generalized ordinal logistic regression model with test as a cluster when the proportionality of odds assumption was not met, as judged by the Wald test described by Williams,^16^ where the significance level was taken to be 5%, was used. The outcome in both cases was the aerosol volume as a three-category variable (0 to <10, 10 to 49, ≥50). First, each characteristic was used in turn as the only fixed effect in the model and then, in the second stage, a model with both technique and container as the fixed effects.

In the second stage, a multivariable model with technique and container as fixed effects was estimated. Volume and titre were both omitted from this model, as they were found to be highly non-significant in the first stage and to avoid having too many parameters in the same model relative to number of observations available for analysis. The *p*-values were obtained by means of the composite Wald test or likelihood ratio test, as appropriate. Measures of effect and their 95% confidence intervals are presented.

## Results

**Figure 1.**
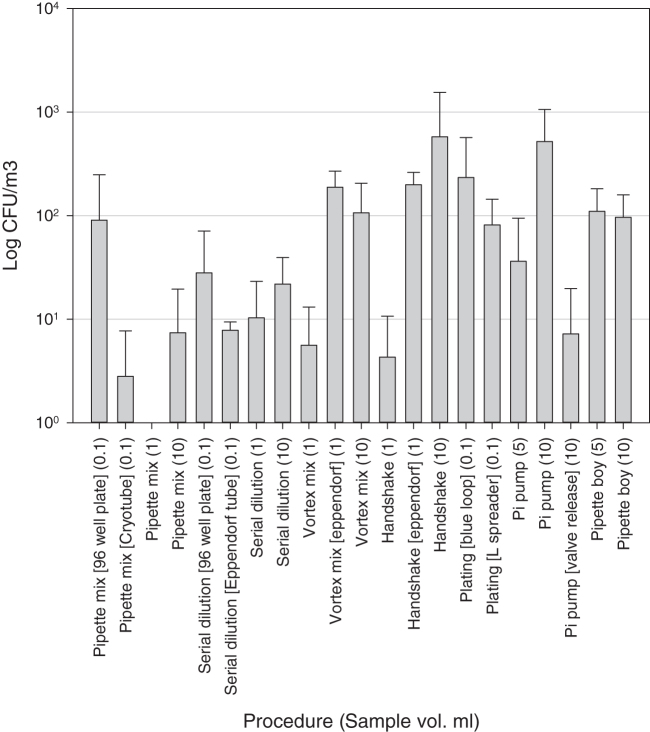
Mean aerosol concentrations generated from the experiments using a 10^9^ cfu/mL spore suspension. All tests were performed in triplicate, except serial dilution 1 mL (*n* = 4) and 10 mL (*n* = 4), vortex mixing universal 1 mL (*n* = 4) and 10 mL (*n* = 4), handshake universal 1 mL (*n* = 4) and 10 mL (*n* = 4), and plating with blue loop and spreader (*n* = 5). Error bars are standard deviations of the means.

Figure 1 shows that the aerosol concentrations generated from the 10^9^ suspension were in the range from 0 to 1563 cfu/m^3^. Plating out the suspension generated aerosols in a range of between 17 and 785 cfu/m^3^. In general, there seems to be a tendency for aerosol concentration to increase as volume handled increased.

**Figure 2.**
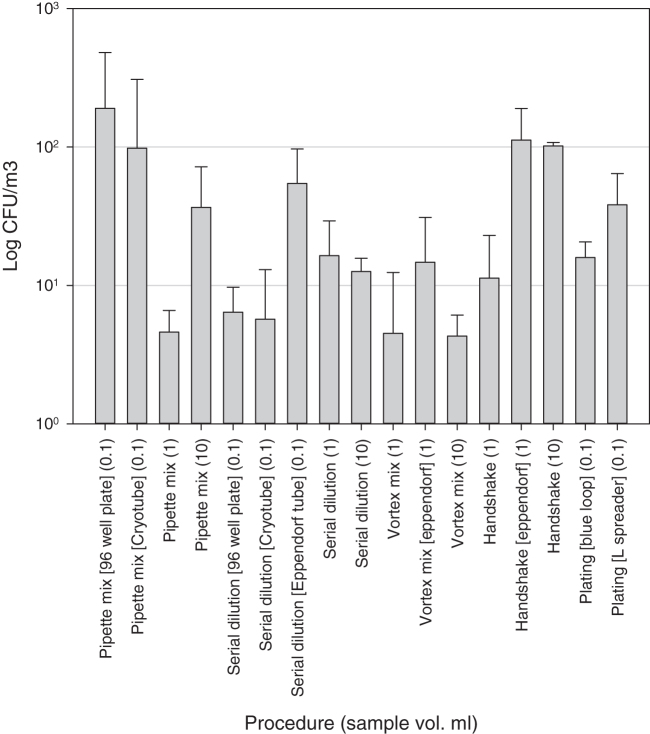
Mean aerosol concentrations generated from the experiments using the 10^7^ cfu/mL spore suspension. *N* = 3 for all tests, and error bars are standard deviations of the means.

Manipulations of the 10^7^ suspension generated aerosols in the range from 0 to 526 cfu/m^3^, with the highest concentrations of aerosols generated from pipette mixing within a 96-well plate and hand shaking volumes of 1 mL in an Eppendorf tube and 10 mL. All other procedures generated aerosol concentrations of <100 cfu/m^3^. Aerosol levels generated from 10^7^ titer suspension were generally lower than those produced from the higher concentration (10^9^) suspension (Figure 2).

**Figure 3.**
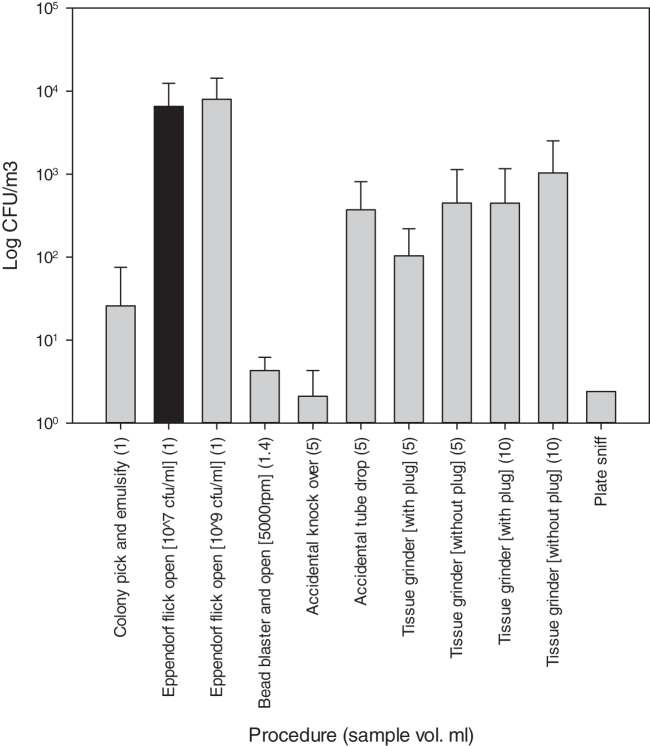
Mean aerosol concentrations generated from the experiments undertaken with poor practice, accidents, and specialist equipment using 10^7^ (black bar) and 10^9^ (gray bars) cfu/mL spore suspensions. *N* = 3 and error bars are standard deviations of the means.

Figure 3 shows the aerosol generated from a range of procedures and accidents. The flick opening of the Eppendorf tubes generated the highest levels of aerosols, up to 10^4^ cfu/m^3^ with both suspension titers; other procedures such as plate sniff, bead blast, and knock over of tube generated levels below background concentrations (<12.9 cfu/m^3^). The results do show a decrease in aerosol production when safety features in the procedures are used, such as tissue grinding with the plug in place.

### Statistical Significance of the Data

The results of this study were analyzed for the statistical significance of aerosol production from different parameters: technique, container, starting titer, and volume. In general, the number of experimental replicates limited the scope of the statistical analysis completed.

The odds of producing more aerosols were increased when the titer was 10^9^ cfu/mL compared with 10^7^ cfu/mL, but these results were not statistically significant (*p* 0.3). Similarly, the same conclusion is reached for higher volumes, but again these results were not statistically significant (*p* 0.3).

A highly significant association (*p* < 0.001) was found between the aerosols generated with container and technique. The use of Eppendorf tubes produces higher odds of more aerosols than the use of 96-well plates, though this was not found to be significant after adjustment for technique. The analysis also reached a similar conclusion for hand shaking compared with pipette mixing, with significance persisting after adjusting for container (*p* < 0.001).

### Risk Assessment

The number of aerosolized micro-organisms being inhaled by an exposed laboratory worker will be dependent on the aerosol concentration, exposure time, the operator's breathing rate, and the use of any containment equipment. This can be expressed in the following equation:

$$D=\left(AC\times t\times BR\right)∕PF,$$

where *D* = dose (cfu), AC = aerosol concentration (cfu/m^3^), *t* = exposure time (min), BR = breathing rate (m^3^/min), and PF = protection factor. The protection factor is the ratio of potential exposure without the containment equipment, divided by the potential exposure with the equipment in place. Table 2 gives a number of example calculations based on the data produced during this study using 10^5^ as the protection factor of a fully functioning BSC^3^ and a breathing rate of 15 L/min (0.015 m^3^/min).^17^

**Table 2.** Calculated potential doses (cfu) a laboratory worker would be exposed to when undertaking procedures on the bench and within a functioning biological safety cabinet

Procedure	Aerosol concentration (cfu/m^3^)	Exposure time (min)	Dose cfu—procedure on an open bench	Dose cfu—procedure within a BSC
Handshake (10 mL) 10^9^	580	10	87	0.00087
Pipette mixing 10^7^ (96-well plate)	190	10	29	0.00029
0.1 mL serial dilution 10^7^ (Eppendorf)	55	30	25	0.00025
Eppendorf flip off	8000	5	600	0.006

BSC, biological safety cabinet.

These calculations can be carried out using any of the data with different variables. In this study, results show that laboratory staff would be exposed to <1 cfu of microbial aerosol when using a correctly functioning BSC, under all tested procedures. However, this assessment does not account for any splashes produced during the procedures.

### Surface Contamination

**Figure 4.**
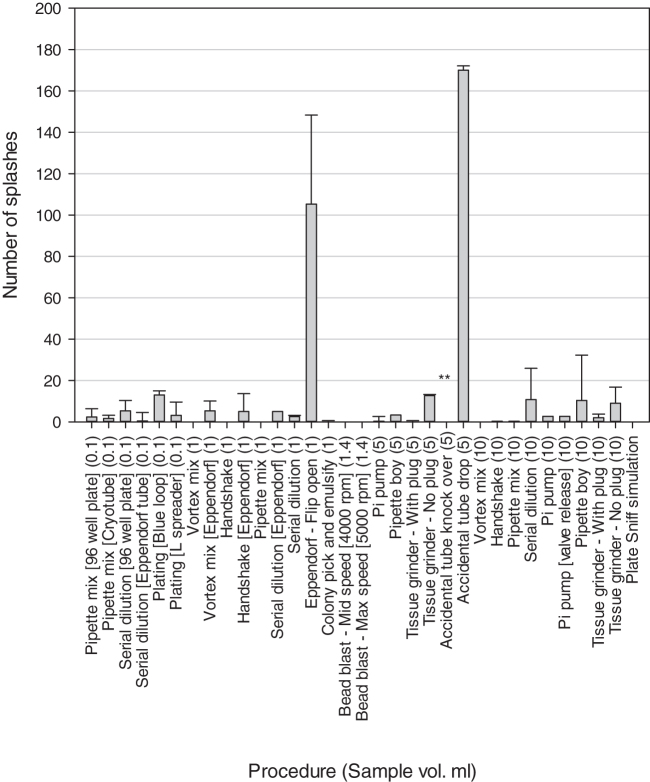
Mean number of splashes counted on the Benchkote immediately under the area where the procedure was completed. ** Indicates that there was a large spillage rather than countable splashes. All tests were completed using the 10^9^ cfu/mL bacterial spore suspension. Error bars are standard deviations of the means.

Figure 4 shows the number of discrete splashes observed after each of the processes was studied, all splashes were counted and not differentiated by size. For most processes, <10 splashes were found. However, processes such as plating out produced higher levels of between 10 and 30 splashes depending on the method and volume used. The highest number of splashes were found from dropping a sample tube (170 splashes) and flipping open the lid of an Eppendorf tube (105 splashes).

## Discussion

The microbial aerosol generated from a range of common processes carried out in the microbiology laboratory has been quantified. It has been shown that a range of aerosols can be generated from the common laboratory processes undertaken within this study. The risk assessment (Table 2) shows that the aerosols generated from every process studied should be contained readily within a functional BSC, without the use of any additional containment measures such as RPE or negative pressure laboratory.

The risk of aerosol exposure to pathogenic agents is measured by the ability of an agent to infect through the respiratory route and the infectious dose of the micro-organisms. Some microorganisms such as *Mycobacterium tuberculosis*, *Coxiella burnetii*, and *Brucella* spp. are reported to have a very low infectious dose (1–10 organisms) by inhalation.^18,19^ However, the vast majority of pathogenic microorganisms are unable to infect through the respiratory route in healthy laboratory staff, unless they are exposed to far higher concentrations of the agent that could overwhelm the lung defenses or potentially cause infection by ingestion after removal from the respiratory tract.^20^

Surface contamination was investigated within this study from the processes tested, and it was found that most procedures give rise to a small amount of surface contamination. Common processes such as plating out generated significant contamination, whereas the highest level of surface contamination was caused by poor practice or accidents. Surface decontamination after microbiological studies have been undertaken and is recommended for all the processes studied.

The level of aerosol generation in the laboratory has been shown to be related to the concentration and volume of the agent handled and also on the training received by the person carrying out the task.^10^ During most of the processes measured, the microbial aerosol concentration collected was <100/m^3^. In all the processes studied, the aerosols generated would have been contained within the cabinet due to the inward directional airflow and there would have been no risk of aerosol exposure to the operator or the laboratory environment.

The results show that generally there is a larger number of aerosols generated from manipulation of the higher concentration suspension (10^9^ cfu/mL) in comparison with the lower concentration suspension (10^7^ cfu/mL). Many of the procedures using the lower concentration suspension generated aerosols of <10 cfu/m^3^ and only three procedures >100 cfu/m^3^. In comparison only six procedures using the higher concentration suspension generated aerosol concentrations <10 cfu/m^3^ and greater than half of the procedures produced 50 cfu/m^3^ or more. As the analysis of the complete data indicates, although not statistically significant, there was a trend that manipulation of higher concentrations generated greater aerosols.

The only exception was pipette mixing 10 mL where the lower concentration suspension generated marginally more aerosols as the higher concentration. The analysis of the data shows that there was an increased likelihood of generating more aerosols when mixing by handshaking tubes in comparison with pipette mixing. This indicates that the use of more controlled mixing with a pipette is an important step in reducing aerosols, an action already taught as part of good laboratory practice.

The results also show that there is a general increase in the number of aerosols with the increase in volumes used for specific procedures. Again, this demonstrates the importance for planning procedures, where if possible smaller volumes should be handled to help reduce aerosols and reduce the risks to the workers. For example, serial dilution 0.1 mL of sample in 0.9 mL diluent rather than 1 mL of sample in 9 mL of diluent. This will help to reduce the aerosols produced and can limit the necessity of primary containment of the procedure.

The time for each procedure was capped at 5 min. This may not reflect all of the working practices in a laboratory, that is, serial dilutions and plating out may take place over a longer time frame, but this has been accounted for in the risk assessment section. Aerosol generation can be reduced by using the correct containers, for example, the use of Eppendorf flip lid tubes generated comparatively high number of aerosols for their volumes used (1 mL) compared with the screw top lidded universal tubes where 10 mL of suspension was used. This was because liquid can be lodged in and around the Eppendorf's lid and the motion of opening the lid can lead to the production of aerosols.

This study has a few limitations. Only three people were used during the study with most of the work being carried out by one individual. But using one person for the majority of the experiments meant procedures were standardized. All the individuals taking part in this study had 1–5 years microbiology laboratory experience, mainly at biosafety level 2. A previous publication has shown that interoperator variation was found among staff carrying out pipetting.^10^ Therefore, one method to reduce aerosol production is to increase worker training in good microbiological practice.

The positioning of the samplers in the study was designed to pick up any aerosol at source. The closeness of the samplers to the process will mean that not just respirable particles were sampled but that larger particles including splashes may also have been collected on the filters. These larger particles would not be respired, and if inhaled would be deposited into the nose or the mouth and subsequently swallowed and passed through the gastrointestinal tract. This means that all the results obtained should be regarded as being an overestimate and that actual operator exposure would be lower. Also, many laboratory agents are not as aerosol stable as *B. atrophaeus* spores and would potentially lose viability during aerosolization and before deposition.^21^

This study only used suspensions of spores in an SDW solution. Aerosol production may differ from other suspending liquids that are found in the laboratory such as blood or sputum. It would be expected that more viscous suspensions would produce less aerosols from the procedures because more energy would be required to disperse the liquid compared with the less viscous SDW suspension. However, viability of microbial aerosols generated from such fluids would persist longer in the environment due to shielding by existing organic material in these fluids.

The study has shown that aerosol generation within the laboratory will be a factor of the process undertaken, the titer and volume of the agent, and the training of the person carrying out the task. In general, every task studied would not cause an aerosol to be released from a normally functioning BSC. Tasks carried out with low volume and low titer agents could be safely carried out on the laboratory bench without containment unless the biological agent handled is predominately transmissible through the aerosol route. None of the processes studied warranted the use of a higher level of containment than a BSC, that is, no need for extra RPE or negative pressure laboratory or terminal HEPA filtration.

Further competency training of laboratory workers can help reduce the aerosols generated during the procedures. This helps to identify the requirement to form a risk-based approach to laboratory safety.^5^ It is hoped that the data provided in this study will be used to inform the risk-based approach set out in the 4^th^ edition of the WHO Laboratory Biosafety manual by providing evidence for quantitative risk assessment. This can be done by using the aerosolization data to allow estimation of the potential aerosol exposure of operators during laboratory procedures and thus informing the choice of an adequate method of aerosol protection.

Air sampling of microbiological processes can be used to gain useful information in risk assessment and in the training of laboratory staff, whereas fluorescein-contaminated solutions can be used to show the importance of addressing surface contamination.

## Supplementary Material

Supplemental data
