# Supplementary material for: Microbial Aerosols Generated from Standard Microbiological Laboratory Procedures
Source: Appl Biosaf. 2022 May 27;27(2):92–9. doi: 10.1089/apb.2021.0038 (PMC9150131; doi:10.1089/apb.2021.0038)
Supplement: Supplemental data [file Supp_DataS1.docx]

Experimental Procedures

The laboratory tasks completed in this work were selected to represent a full range of procedures carried out in laboratories globally. Some tasks were small parts of a larger procedure, such as pipette mixing, whilst other experiments encompassed a full procedure, e.g. serial dilution. Some short tasks were carried out multiple times within the 5 minute period which may not reflect normal use but can be used to understand aerosol generation per procedure by standardising the time. Other procedures have been studied using different tires and volumes.

Mixing tests

Three different methods of mixing were studied: a) vortex mixing, b) hand shaking, and c) pipette mixing. For vortex mixing tests, 10 cryo-tubes (Sarstedt, UK) or 30 ml universal tubes (Sterilin, UK) filled with 1 ml and 10 ml, respectively, of a 10^7^ or 10^9^ cfu/ml *Bacillus atrophaeus* suspension with additional fluorescein, were each vortexed for 15 seconds, lids opened immediately for 5 secs, closed and repeated, with 10 replicates per run. For hand shaking tests, cryo-tubes and universals with the same volumes and titres described previously were each vigorously shaken 30 times at a ~45^o^ angle in an up and down motion, lids opened immediately for 5 secs, closed and repeated, with 10 replicates per run. In pipette mixing tests, 0.1 ml, 1 ml and 10 ml volumes in cryo-tubes, cryo-tubes and universals respectively, with a 10^7^ and or 10^9^ cfu/ml suspensions, were each mixed by 10 pipette plunger strokes (1 ml stripette with Pipetboy (Integra, UK) used for 10 ml samples) using 0.05 ml, 0.1 ml and 1 ml aliquot volumes respectively; after which the lid was replaced and repeated for each 10 samples for one run. Pipette mixing of 0.1 ml sample volume was also carried out in 96 well microtiter plate (Nunc, VWR, UK) using 5 rows at a time with multichannel micropipette for 10 wells (equalling 50 wells in total).

Serial dilution tests

In serial dilution tests, 0.1 ml, 1 ml and 10 ml sample volumes in Eppendorf tubes (Eppendorf, UK), cryo-tubes and universals tubes, respectively, separately at both 10^7^ and 10^9^ cfu/ml titre were serially diluted by transfer of 0.05 ml aliquot for 0.1 ml sample; 0.1 ml aliquot for 1 ml sample; and 1 ml aliquot for 10 ml sample (1 ml stripette with Pipetboy) across 11 sample tubes to complete a total of 10 replicates per run. Vortex mixing (Ika Vortex, Genius 3, VWR, UK) between samples was completed for a maximum of 3 sec to reduce this mixing influence on results. Serial dilution of 0.1 ml sample volume was also carried out in 96 well microtiter plate using 5 rows at a time with multichannel micropipette for 10 wells (equalling 50 wells in total)

Plating out

Two bacterial suspension volumes of 0.1 ml was spread on 10 Trypticase Soy Agar plates (TSA, Oxoid, UK) using a 10 μl blue plastic loop (Nunc, UK) or standard spreader (hockey stick). Initial tests were performed with the 10^9^ cfu/ml suspension for both sample volumes and the test repeated with 10^7^ cfu/ml for 0.1 ml aliquot, in each case using blue loops or hockey sticks.

Large volume pipetting

Large volume pipetting tests were performed using a 5 ml and 10 ml volume of a 10^9^ cfu/ml spore suspension into 10 universal tubes per run. Two modes of liquid transfer into each tube was tested i.e. manual piston operated pipette filler (Pi-Pump, SLS, UK) with thumb-wheel filling and emptying control release valve level for rapid emptying, and a battery powered Pipetboy. In each case the liquid was dispensed with a 10 ml stripette 1/4 of the way down the tube’s sidewall, pressing down the Pi-Pump plunger with the thumb for quick dispensing, or dispensing speed set between low to medium setting for the Pipetboy. Residual liquid was purged by reeling up the Pi-Pump thumb-wheel half a turn and pressing down the plunger again once, while a 2 second pause was allowed after initial dispensing with Pipetboy followed by a single press on the dispensing trigger button. The dispensing of 10 ml suspension with the Pi-Pump was repeated under more controlled technique using only the control release valve for comparison to the worst-case scenario described above.

Tissue grinder homogenisation

Homogenisation with a tissue grinder was simulated using 5 ml and 10 ml volumes of a 10^9^ cfu/ml spore suspension in the glass tube. The pestle rode had a plug (stopper) that could be lowered into place to create a seal at the top of the glass tube. Tests were performed with and with-out the plug in place to simulate open and closed manipulation procedures and ascertain the difference in aerosol concentration release. The pestle grinder was pushed up and down 20 times in a slow twisting motion each stroke, with the tube held vertically with aid of a rack. For this test, the MD8 sampler heads were set up at 23 cm height from the cabinet floor to capture the aerosol release at source.

Eppendorf tube procedures

One millilitre sample aliquots of 10^7^ and or 10^9^ cfu/ml were used for sample manipulation in snap-top (Eppendorf) tubes. In flipping lid open tests, 10 tubes per run were briefly vortexed to allow suspension deposits in the lids, allowed aerosols to settle and flicked open with a thumb at ~70o – 80o angle. Vortex mixing and hand shake mix was also performed as previously described, and 0.1 ml aliquots were serially transferred across 11 tubes.

Bead blast homogenisation

Blead blast tissue homogenisation simulation was carried out using the Bertin Minilys (Bertin Techonologies, Streton Scientific, UK) and Minilys/Precellys lysing kit (2 ml tubes). Three Minilys tubes (with O-rings) containing ceramic beads were filled with 1.4 ml 10^9^ cfu/ml suspensions, tubes were then loaded into the Minilys unit positioned within the Class II cabinet. An initial cycle was run at medium speed (4000 rpm) for 180 sec while air was sampled around the unit at the same time. At the end of the 180 sec cycle each tube was removed and lids immediately opened for 5-10 secs as a worst case scenario. Lids were replaced and air sampling left to run for the total 5 mins. The test was repeated at maximum speed of 5000 rpm as a comparison. For this test one MD8 sampler was position 5 cm above the Minilys unit’s front cover facing down at a small opening gap as potential aerosol escape route, and the other MD8 at the front of the unit directly in front of the cover handle opening gap.

Accidental spill

In accidental spill tests, a universal tube containing 5 ml of 10^9^ cfu/ml suspension was used. Two accidental spill scenarios were tested; a) knocking over a tube at cabinet floor level, and b) dropping a tube from a height of 18 – 20 cm. Tests were undertaken in a Class III BSC to contain potential excess splashes and aerosols from dropping of tubes, whilst the fan units were not running.

Plate sniffing simulation

A 100 μl suspensions of *B. atrophaeus* spores at 10^9^ cfu/ml was spread on 5 x TSA plates for lawn bacterial growth. After 24 hr incubation, a TSA plate was positioned under a down facing Sartorius MD8 filter head with a 2.5 cm clearance and re-placed with a fresh plate at 1 min intervals whilst the air was sampled continuously for 5 min.

Colony pick and emulsification

For colony pick an emulsification, single colonies of B*. atrophaeus* spores were grown on TSA and using a 10 μl blue loop a single colony was picked and resuspended in 1 ml SDW in an Eppendorf tube, with vigorous twisting of the loop for 2 min while air was sampled and tube left open for the complete 5 min sampling.

Participants

Initial tests were completed by one individual with 5+ year laboratory experience, after which two volunteers, one male and one female, with less than 1 year lab experience were recruited from existing research staff members at Public Health England, to undertake a select number of the above test procedures commonly carried out in the lab setting worldwide. Each volunteer was provided with a standard test protocol to follow under controlled conditions, thus their current lab experience was not critical for this study. The test procedures carried out were serial dilution of 0.1 ml, 1 ml and 10 ml sample volumes in Eppendorf, cryo-tubes and universals respectively; and standard plate out of 0.1 ml suspension on TSA, spread with hockey sticks as previously described above. Both tests were undertaken with 10^7^ and 10^9^ cfu/ml spore suspension suspensions.

All tests were performed with a view of worst case scenario unless stated otherwise and in triplicate independent runs.
